# Nested PCR detection of malaria directly using blood filter paper samples from epidemiological surveys

**DOI:** 10.1186/1475-2875-13-175

**Published:** 2014-05-08

**Authors:** Peipei Li, Zhenjun Zhao, Ying Wang, Hua Xing, Daniel M Parker, Zhaoqing Yang, Elizabeth Baum, Wenli Li, Jetsumon Sattabongkot, Jeeraphat Sirichaisinthop, Shuying Li, Guiyun Yan, Liwang Cui, Qi Fan

**Affiliations:** 1 Dalian Institute of Biotechnology, Dalian, Liaoning, China; 2 Institute of Tropical Medicine, Third Military Medical University, Chongqing, China; 3 Dalian University of Technology, Dalian, Liaoning, China; 4 Department of Entomology, Pennsylvania State University, University Park, Pennsylvania, PA, USA; 5 Department of Pathogen Biology and Immunology, Kunming Medical University, Kunming, Yunnan, China; 6 Program in Public Health, University of California, Irvine, CA, USA; 7 Faculty of Tropical Medicine, Mahidol University, Bangkok 10400, Thailand; 8 Vector Borne Disease Training Center, Pra Budhabat, Saraburi 18120, Thailand

**Keywords:** Malaria, PCR detection, Blood filter paper, Epidemiology, Sensitivity, Accuracy

## Abstract

**Background:**

Nested PCR is considered a sensitive and specific method for detecting malaria parasites and is especially useful in epidemiological surveys. However, the preparation of DNA templates for PCR is often time-consuming and costly.

**Methods:**

A simplified PCR method was developed to directly use a small blood filter paper square (2 × 2 mm) as the DNA template after treatment with saponin. This filter paper-based nested PCR method (FP-PCR) was compared to microscopy and standard nested PCR with DNA extracted by using a Qiagen DNA mini kit from filter paper blood spots of 204 febrile cases. The FP-PCR technique was further applied to evaluate malaria infections in 1,708 participants from cross-sectional epidemiological surveys conducted in Myanmar and Thailand.

**Results:**

The FP-PCR method had a detection limit of ~0.2 parasites/μL blood, estimated using cultured *Plasmodium falciparum* parasites. With 204 field samples, the sensitivity of the FP-PCR method was comparable to that of the standard nested PCR method, which was significantly higher than that of microscopy. Application of the FP-PCR method in large cross-sectional studies conducted in Myanmar and Thailand detected 1.9% (12/638) and 6.2% (66/1,070) asymptomatic *Plasmodium* infections, respectively, as compared to the detection rates of 1.3% (8/638) and 0.04% (4/1,070) by microscopy.

**Conclusion:**

This FP-PCR method was much more sensitive than microscopy in detecting *Plasmodium* infections. It drastically increased the detection sensitivity of asymptomatic infections in cross-sectional surveys conducted in Thailand and Myanmar, suggesting that this FP-PCR method has a potential for future applications in malaria epidemiology studies.

## Background

Malaria, causing approximately 660,000 deaths each year, remains a major global health problem [1]. In the last decade, with substantially increased funding and more effective management through long-lasting insecticidal nets, rapid diagnostic tests (RDTs) and artemisinin-based combination therapy (ACT), malaria control has achieved encouraging results and malaria elimination has attracted increasing attention. The elimination of malaria transmission requires enhanced surveillance and control efforts, which rely on rapid detection and effective treatment of infections. This is especially important with regard to asymptomatic parasite carriers, which could serve as important reservoirs for continued transmission [2,3]. Most national malaria control programmes in endemic countries have installed passive case detection (PCD), whereas some also conduct active case detection (ACD) at sentinel sites. Currently, malaria case detection depends heavily on microscopy and/or RDTs. However, both methods miss infections when parasite densities are low (<10 parasites/μL) [4]. These submicroscopic infections are quite common in areas where malaria is seasonal and unstable [3,5,6], and they, therefore, represent major challenges for malarial control and elimination programmes.

Compared with conventional microscopy or RDTs, molecular tools, such as PCR, are highly sensitive in detecting low-density infections and determining the parasite species [7]. Although PCR has limitations for use as a routine diagnostic method in clinical settings, it has been used extensively in malaria surveillance. For this purpose, samples can be pooled for DNA extraction and analysis to further reduce the cost and increase the throughput [8-10]. For PCR, a routinely used method for parasite DNA extraction from dried blood spots is the Chelex boiling method [11], which increases the chances of sample cross contamination when many samples are processed at the same time. In our experience, DNA samples obtained using the Chelex method tend to degrade faster than those obtained using the Qiagen kit after extended storage at −20°C. Alternatively, filter paper blood spots are directly used in PCR without extraction [12]. Yet, washing filter blood spots with warm water during this procedure may lead to the loss of parasite DNA. In this report, a procedure to include a saponin treatment step of filter paper blood samples for direct PCR analysis was developed. It was further validated for malaria surveillance using samples from PCD and cross-sectional studies conducted in western Thailand and north-east Myanmar.

## Methods

### Study areas and sample collection

This study was performed at study sites in northeast Myanmar and western Thailand, where malaria is characterized as hypoendemic and seasonal. The study areas have a predominance of *Plasmodium falciparum* and *Plasmodium vivax* infections. For method development and initial validation, samples from 204 randomly selected febrile patients (temperature >37.5°C, 112 males and 92 females, ages ranging from six months to 73 years) suspected of uncomplicated malaria at malaria clinics near Laiza township, Kachin State in north-east Myanmar were evaluated. This method was further applied to 1,708 samples collected from cross-sectional studies, including 638 participants recruited from 10 villages near Laiza township in May and August of 2011, and 1,070 samples collected in four border villages of Tha Song Yang district, Tak province, western Thailand in August and December of 2011 and May of 2012. Finger-prick blood samples were collected onto Whatman 3 M filter paper, air dried, and individually placed in plastic bags for storage. Informed consent/assent was obtained from each participant. The study protocol was approved by institutional review boards at the Pennsylvania State University, the Thai Ministry of Health, and Kunming Medical University, China.

### Microscopy

Thick and thin blood films were prepared from peripheral blood. The slides were stained with Giemsa and screened for malaria parasites by microscopy with (100×) oil immersion lens. Blood films were examined by two microscopists with at least five years of experience in malaria microscopy, who were blinded to each other’s result. Smears were considered negative if no parasite was seen in 100× oil immersion fields in a thick blood film.

### Sample preparation

For the standard PCR method, DNA was extracted from ~100 μL of dried blood on filter paper using the Qiagen DNA Mini Kit following the manufacturer’s recommendation. DNA was eluted in 50 μL of H_2_O. For the direct PCR method from filter paper (FP-PCR), a 2 × 2 mm square was punched from blood spot into a 1.5 mL centrifuge tube. One hundred microlitres of 0.5% saponin in phosphate-buffered saline (PBS, pH 7.0) were added and incubated at room temperature for 10 min, and then were inverted a few times to mix. The samples were centrifuged at 13,000 × g for 1 min, and the supernatant was removed and discarded. Then the filter papers were washed with 1 mL of PBS, briefly dried and used immediately for PCR.

### PCR

A modified nested PCR amplification was performed as described previously based on small subunit rRNA gene [12-14]. For the primary standard PCR reaction, 2 μL of genomic DNA were used in a 25 μL reaction with outer primers rPLU5 and rPLU6 [15]. For the FP-PCR, the same 25 μL reaction was prepared with DNA template replaced by the filter paper disk. PCR was done under the same cycle conditions, which included an initial denaturing period at 94°C for 4 min and 30 cycles of 94°C for 1 min, 55°C for 2 min and 72°C for 2 min, and a final extension for 5 min. Nested PCR was performed with 2 μL of the primary PCR product and species-specific primers for the four human malaria species and *Plasmodium knowlesi* in separate reaction tubes. Thirty PCR cycles (94°C for 40 s, 58°C for 1 min and 72°C for 2 min) were performed [13].

For blood filter samples collected during cross-sectional studies, the primary FP-PCR was done using the same conditions with blood filter paper discs as DNA templates and outer primers rPLU1 and rPLU5. Because a small fraction of the blood filter samples were diagnosed for malaria, nested PCR was done using genus-specific primers rPLU3 and rPLU4 under the same cycle conditions except for the change of annealing temperature to 62°C [16]. For the PCR positive samples, nested PCR was performed using species-specific primers for the four human malaria species and *P. knowlesi* in separate reaction tubes.

All PCR products were separated in 1% and 2% agarose gels for primary and nested PCR, respectively. After staining with ethidium bromide, the gel was visualized under a UV light.

### Detection limit of the FP-PCR

To determine the sensitivity of the FP-PCR, *P. falciparum* 3D7 parasites were cultured in type O^+^ RBCs in complete medium supplemented with 10% human AB serum under an atmosphere of 90% N_2_/5% O_2_/5% CO_2_[17]. Synchronization of parasite cultures with 5% sorbitol was performed as previously described [18]. Ring stage parasites at 2% parasitaemia were concentrated to ~45% haematocrit. Two-fold serial dilutions were performed to the lowest parasitaemia of 1.7 × 10^−12^% by adding an equal volume of uninfected RBCs at the same haematocrit. One hundred microlitres of each dilution were spotted on Whatman 3 M filter paper, air-dried and stored at −20°C until use. From each spot, ten 2 × 2 mm squares were individually used for FP-PCR analysis as described above or the original filter paper blood PCR method [12]. Parasite density was estimated by using an RBC count of 5 million/μL blood.

### Data analysis

Diagnostic performance for each test was evaluated with the results of nested PCR method using DNA extracted from blood spots by using the Qiagen kit. Performance indices were the number of true positive (TP), the number of true negative (TN), the number of false positive (FP) and number of false negative (FN). Sensitivity was expressed as TP/(TP + FN) and specificity as TN/(TP + FP). Accuracy of the tests were calculated as (TP/TN)/number of all tests. *Kappa* statistics were used to compare the agreement against which might be expected by chance, with possible values ranging from 1 (perfect agreement) to – 1 (complete disagreement) and 0 indicating agreement attributable to chance.

## Results

### The FP-PCR method

In order to avoid potential cross contamination due to heating during DNA preparation from filter paper blood [12], we evaluated a nested PCR method using 2 × 2 mm squares of blood filter paper directly after simple saponin treatment and buffer washing. Saponin treatment lyses cholesterol-rich RBC membranes and subsequent buffer washing removes PCR inhibitors such as hemoglobin. To test the feasibility of this procedure, we first compared this FP-PCR method with the original filter paper blood PCR method [12] and standard PCR protocol using DNA isolated with the Qiagen kit. The blood filter paper was prepared from serial dilutions of *P. falciparum* laboratory culture with a known parasitemia. Under these experimental conditions, the lowest parasitaemia that consistently produced positive PCR result for the two filter paper methods was 3.8 × 10^−6^%, corresponding to ~0.2 parasites/μL blood. In comparison, the lowest parasitaemia detected for the PCR using DNA prepared by the Qiagen kit was 3.1 × 10^−5^%, corresponding to ~1.5 parasites/μL.

### Validation of the FP-PCR method using field samples

The suitability of the FP-PCR method for malaria surveillance was evaluated using 204 febrile cases showing malaria-like symptoms from a malaria clinic in northeastern Myanmar. The FP-PCR method was compared directly with microscopy and standard PCR method using DNA extracted with the Qiagen kit. Microscopic examination of blood films by two experienced microscopists detected malaria parasites in 169 patients, including 58 *P. falciparum*, 93 *P. vivax*, and 18 *P. falciparum/P. vivax* mixed infections (Table 1). Compared with microscopy, both nested PCR methods displayed higher sensitivities. Standard nested PCR using DNA prepared with the Qiagen kit detected 179 malaria cases infected with *P. falciparum* (n = 68), *P. vivax* (n = 83), and both species (n = 28) (Table 1). Similarly, the FP-PCR method detected 178 malaria cases infected with *P. falciparum* (n = 78), *P. vivax* (n = 93) and both species (n = 7) (Table 1). If nested PCR with kit prepared DNA was used as the gold standard, the sensitivity of microscopy for detection of *P. falciparum* and *P. vivax* was 74.0 and 82.9%, respectively, whereas the sensitivity of the FP-PCR method for detecting *P. falciparum* and for *P. vivax* was increased to 88.5 and 90.1%, respectively. Correspondingly, the test accuracy of microscopy for *P. falciparum* and *P. vivax* was 85.3% and 81.4% respectively, which was increased to 94.6% for both parasites by the FP-PCR method (Table 2). With clearly increased sensitivity, the standard PCR method improved the detection sensitivity and accuracy of mixed species infections. Although microscopy detected 18 mixed species infections, only two were confirmed by the standard PCR method. Somehow, the FP-PCR method was less sensitive in detecting mixed species infections. Of the 28 mixed infections detected by the standard PCR method, the FP-PCR method only confirmed 7 as mixed infections, whereas the remaining 21 were determined to be 10 *P. falciparum* and 11 *P. vivax* single infections.

**Table 1:** Comparison of diagnostic results from microscopy and two nested PCR results in 204 PCD cases

**Method**	**Result**	**Nested PCR (using DNA purified by kit)**				**Total**
		***P. falciparum***	***P. vivax***	***Mix of Pf & Pv***	***Negative***	
Microscopy	*P. falciparum*	45	2	10	1	58
	*P. vivax*	4	74	14	1	93
	*Mix of Pf & Pv*	14	2	2	0	18
	*Negative*	5	5	2	23	35
Nested PCR (using blood filter papers)	*P. falciparum*	68	0	10	0	78
	*P. vivax*	0	82	11	0	93
	*Mix of Pf & Pv*	0	0	7	0	7
	*Negative*	0	1	0	25	26
	Total	68	83	28	25	204

**Table 2:** Performance of microscopy and FP-PCR method in 204 PCD cases

**Method/Specimen**	**Category**	Sensitivity (%)^*^		**Accuracy (%) (n = 204)**	***Kappa *(n = 204)**
		**Total 204 samples**	**176 samples exclude mix infected cases**		
Microscopy	*P. falciparum*	74.0 (65.7-82.2)	86.8 (80.4-93.2)	85.3	0.70
	*P. vivax*	82.9 (75.2-90.5)	91.6 (85.9-97.2)	81.4	0.63
FP-PCR	*P. falciparum*	88.5 (82.5-94.6)	100.0 (100.0-100.0)	94.6	0.89
	*P. vivax*	90.1 (84.0-96.2)	98.8 (96.6-100)	94.6	0.89

^*^Data are presented as percentage (95% confidence interval; CI). The results from nested PCR with DNA prepared by the Qiagen kit were used as the gold standard.

### Applications of FP-PCR technique in epidemiological surveys

The FP-PCR method was further validated using 1,708 filter paper blood samples collected from several cross-sectional surveys. Of the 638 samples from northeastern Myanmar, microscopy detected eight malaria cases (two *P. falciparum*, three *P. vivax* and three *P. falciparum*/*P. vivax* mixed infections). FP-PCR confirmed the two *P. falciparum* and three *P. vivax* cases. For the three mixed infections, FP-PCR identified them as one *P. falciparum* single-species infections and two negative cases. In addition, FP-PCR detected an additional one *P. falciparum* and five *P. vivax* infections from the microscopy-negative cases (Table 3).

**Table 3:** Performance of microscopy and FP-PCR technique in cross-sectional surveys

**Sites**	**Methods**	***P. falciparum***	***P. vivax***	***P. falciparum + P. vivax***	***P. malariae***	***P. ovale***	***Negative***	**Total**	**Positive rate**
Myanmar	FP-PCR	4	8	0	0	0	626	638	1.9%
	Microscopy	2	3	3	0	0	630	638	1.3%
Thailand	FP-PCR	13	48	4	1	0	1,004	1,070	6.2%
	Microscopy	1	3	0	0	0	1,066	1,070	0.04%

Of the 1,070 samples from three cross-sectional studies in western Thailand, only four cases (one *P. falciparum* and three *P. vivax*) were initially identified by microscopy. In contrast, FP-PCR detected a total of 66 malaria infections (13 *P. falciparum*, 48 *P. vivax*, one *Plasmodium malariae*, and four mixed species infection by *P. falciparum* and *P. vivax*) (Table 3). The *P. malariae* case was verified by sequencing of the PCR product. It is worth to mention that a subset 217 Thai samples collected in May 2012 were detected by FP-PCR with a malaria infection rate of 13.8%. This infection rate largely agreed with the results obtained by PCR using DNA prepared by the Qiagen kit using filter paper blood samples collected in the same village at the same time.

## Discussion

Microscopy and RDTs both underestimate malaria parasite prevalence, especially for asymptomatic infections with a low parasitemia. Here a FP-PCR method for the detection of malaria parasites in dried blood filter paper was described. This PCR method uses a small square of blood filter paper directly as the source of parasite DNA, which significantly reduces the time and cost of DNA preparation. It incorporated a simple step of saponin treatment of the blood filter paper square in order to lyse the RBCs and remove potential inhibitors for PCR. While this FP-PCR method was similar in sensitivity to the original filter paper PCR method [12], the FP-PCR method does not require a heated step during DNA extraction, thus reducing the chances of potential cross contamination. The detection limit of the FP-PCR method determined using cultured *P. falciparum* parasites was approximately 0.2 parasites/μL blood, ~50 times more sensitive than the detection limit of >10 parasites/μL blood of microscopy. Yet, the FP-PCR method underperformed on mixed species infections when compared with the Qiagen kit method using clinical samples. With parasite samples from PCD, the FP-PCR method was much more sensitive than microscopy. The simplified DNA preparation involved in the FP-PCR suggests a good potential for applications in epidemiological studies of malaria.

With a large sample collection of blood filter papers from cross-sectional surveys in the Thailand and Myanmar, the FP-PCR method was found to drastically increase the detection sensitivity over conventional microscopy, especially for asymptomatic infections. One of the current malaria management strategies at these sites focuses on timely treatment of malaria cases with appropriate anti-malarial drugs, aiming at reducing and eliminating malaria transmission from these active cases. However, the presence of large number of asymptomatic infections in the study population that went undetectable by microscopy suggests that treating the symptomatic cases alone will not completely stop malaria transmission. These asymptomatic malaria parasite carriers may serve as important sources of continued malaria transmission in these border regions. It underlines the significance of deploying more sensitive methods of parasite detection of the asymptomatic infections, which will enable the planning and execution of radical measures such as mass drug administration for malaria elimination.

## Conclusion

A FP-PCR method for the detection of malaria parasites using a small square of blood filter paper directly as the source of parasite DNA was established. This method significantly reduces the time and cost of DNA preparation. It was much more sensitive than microscopy. The performance in cross-sectional surveys in the Thailand and Myanmar showed that the FP-PCR method drastically improved the detection of asymptomatic infections.

## Competing interests

The authors declare that they have no competing interests.

## Authors’ contributions

PL, ZZ, YW, HX and ZY carried out the experimental work and data analysis. WL, DMP, ZY, EB, JS and JS participated in data analysis. PL performed manuscript writing. GY, LC and QF conceived the study and participated in the design of the study. All authors read and approved the final manuscript.
